# Development and evaluation of loop-mediated isothermal amplification assay for rapid detection of *Capripoxvirus*

**DOI:** 10.14202/vetworld.2015.1286-1292

**Published:** 2015-11-05

**Authors:** Kanisht Batra, Aman Kumar, Vinay Kumar, Trilok Nanda, Narender S Maan, Sushila Maan

**Affiliations:** 1 Department of Animal Biotechnology, College of Veterinary Sciences, LLR University of Veterinary and Animal Sciences, Hisar, Haryana, India; 2 Resource Faculty, Department of Animal Biotechnology, College of Veterinary Sciences, Lala Lajpat Rai University of Veterinary and Animal Sciences, Hisar, Haryana, India

**Keywords:** *Capripoxvirus*, loop-mediated isothermal amplification assay, real-time polymerase chain reaction, sensitivity, specificity

## Abstract

**Aim:**

The present study was undertaken to develop a nucleic acid-based diagnostic assay loop-mediated isothermal amplification assay (LAMP) targeting highly conserved genomic regions of Capripoxvirus (CaPVs) and its comparative evaluation with real-time polymerase chain reaction (PCR).

**Material and Methods:**

Lyophilized vaccine strain of sheeppox virus (SPPV) was used for optimization of LAMP assay. The LAMP assay was designed using envelope immunogenic protein (P32) coding gene targeting highly conserved genomic regions of CaPV responsible for causing sheep pox, goat pox, and lumpy skin disease in sheep, goat and cattle respectively. Serial tenfold dilution of SPPV recombinant plasmid DNA was used for a calculating limit of detection. Analytical sensitivity and specificity were performed.

**Results:**

The test described is quick (30 min), sensitive and specific for detection of CaPVs. The described assay did not show any cross-reactivity to other related viruses that cause apparently similar clinical signs. It was found to be ten times more sensitive than conventional PCR however, 100 times less sensitive than quantitative PCR (qPCR). LAMP assay results were monitored by color change method using picogreen dye and agarose gel electrophoresis.

**Conclusion:**

LAMP assay can be a very good alternative for CaPV detection to other molecular techniques requiring sophisticated equipments.

## Introduction

Diseases caused by Capripox viruses (CaPVs) are among the most serious pox viral diseases of ruminants. The causative agent is double stranded DNA virus belonging to genus *Capripoxvirus*, subfamily *Chordopoxvirinae*, family *Poxviridae* including three closely related species, i.e., goatpox virus (GPPV), sheeppox virus (SPPV), and lumpy skin disease virus (LSDV). They alone are responsible for significant economic losses in endemic countries. Due to their nature of serious and rapid spread they are listed as notifiable diseases by World Organisaton for Animal Health (OIE) [1]. SPPV and GPPV are endemic in Indian sub-continent, North Africa, China, Turkey and Middle East. Lumpy skin disease is confined to sub-Saharan African countries, Egypt and Israel [1,2].

In Maharashtra state of India alone, losses due to SPPV and GPPV (with an average morbidity and mortality) are estimated over INR 107.5 million and annual loss at the national level extrapolates to INR 1250 million [3]. This disease can be associated with significant production losses due to increased abortion rates, damage to wool, decreased milk production and increased susceptibility to pneumonia, leading to mortality. The large-scale economic losses due to this disease can justify its threat as a potential bioterrorism agent.

There are varieties of available vaccines such as gel absorbed vaccines, live attenuated vaccines polypeptide, and combine vaccines, in which single strain of CaPV can provide immunity to both sheep and goat [2]. Although a live attenuated vaccine is available and being used [4], there have been several reports of disease in sheep and goat from different parts of India [5-7].

For controlling any outbreak, the foremost requirement is the rapid, sensitive, specific, and robust tool for diagnosis of the causative agent. Although various serological techniques, such as agar gel precipitation test, counter-immunoelectrophoresis [8, 9], indirect ELISA, and virus neutralization test, are available for diagnosis of these diseases; these tests have certain limitations such as low antibody response, time-consuming tissue culture isolation, and low specificity (based on their cross reactions with Orf virus-a Parapoxvirus) [10,11].

Nucleic acid-based techniques like gel based polymerase chain reaction (PCR) assay, real-time PCR are either more expensive or requires well-equipped laboratory [11-19]. Loop-mediated isothermal amplification assay (LAMP) has highly specific DNA dependent amplification using four to six pair of primers targeting six to eight genomic regions [20]. A highly conserved P32 envelope gene was targeted for designing LAMP for CaPV. This gene is highly suitable for discrimination of animal origin viruses and can detect all CaPVs. There is rapid strand displacing activity of DNA polymerase and isothermal amplification of LAMP which allows it to take place within half an hour at a temperature between 60 and 65°C. LAMP assay have been successfully applied in lateral flow devices format [21] which involves conjugation of forward and reverse internal primers with fluorophore. This provides a potential simple-to-use tool for field based detection of the virus.

Therefore, exploiting the above features of the LAMP assay this study was conducted to develop a LAMP assay based on highly conserved P32 envelope gene for the simultaneous detection of CaPVs.

## Materials and Methods

### Ethical approval

The samples used in this study were collected from naturally infected/dead animals in the field, by qualified veterinarians, as part of routine diagnostic, hence ethical approval was not necessary.

### Virus isolation and isolates

A lyophilized Indian vaccine strain (Rumanian Fanar) of SPPV was procured from Haryana Veterinary Vaccine Institute, Hisar. The vaccine virus was reconstituted in 1 ml phosphate buffer saline (PBS) and was used to infect primary lamb testis cell culture. Lamb testicle cells were grown in minimal essential media M199 (Sigma-Aldrich, USA) supplemented with L-glutamine, sodium bicarbonate at 0.35 g/liter, antibiotics (100 µg streptomycin and 100 IU penicillin/100 ml) and 10% fetal bovine serum (Life Tech). After 3 days post-infection, infected cells showing >80% cytopathic effect (CPE) were harvested, and cell pellets were used for isolation of viral DNA.

### DNA extraction

The infected cells were pelleted down by centrifugation at 573 × *g* for 15 min. The pellets were then resuspended in 250 μL of PBS and DNA was extracted using PureLink™ Genomic DNA isolation Kit (Invitrogen) according to manufacturer’s instruction. This is based on the selective binding of DNA to silica-based membrane in the presence of chaotropic salts. The extracted DNA was eluted in 30 μl of PureLink™ Genomic elution buffer provided with the kit. Quantitative analysis of purified DNA was performed using Qubit^®^ 2.0 Fluorometer (Invitrogen).

### Cloning of partial P32 gene

Genomic DNA from virus isolate of SPPV was subjected to PCR using the primers (CaPV-P32/Terminal Forward 5’-ATGGCAGATATCCCATTATATGTTA-3’; CaPV-P32/Internal Reverse: 5’-GACGATAATCTAAT TACATATG-3’) to amplify 578 bp portion of P32 gene [22]. The PCR amplicon was cloned into CloneJET™ PCR Cloning Kit (Thermo Scientific K1230). The recombinant plasmids were purified and sequenced with the BigDye Terminator Cycle Sequencing Kit (Applied Biosystems, Carlsbad, CA, USA) on an automatic ABI 3130 xl Genetic Analyzer (Applied Biosystems, Carlsbad, CA, USA). The sequencing data were analyzed with Lasergene ver 5.0 software (Invitrogen, Carlsbad, CA, USA).

### LAMP primer design

**Figure-1:**
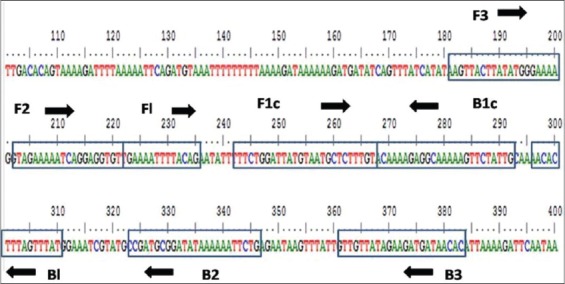
Location of loop mediated isothermal amplification assay primers along the P32 gene sequence.

Oligonucleotides targeting conserved P32 gene of SPPV, GPPV, and LSDV were designed using Primer Explorer V4 software (Fujitsu System Solutions) (Table-1). Different SPPV, GPPV, and LSDV sequences available in the database were aligned and *in silico* analysis were done for alignment with homologues of other poxviruses such as vaccinia virus and contagious pustular dermatitis (Orf) virus to achieve a higher level of specificity using BioEdit version 7.1.3. To account for an AT-rich template, software settings were changed including selecting a lower melting temperature (Tm), increased primer length and shorter distance between primers. Figure-1 shows the position of various LAMP primers from nucleotide position 181-383 in P32 gene.

**Table-1:** LAMP primers used for amplification of CaPV.

Primer name	Length (nucleotides)	Genome positions	Sequence (5’-3’)
FIP			
F1c	41	243-267	ACAAAGAGCATTACATAATCCAGAA-
F2		203-221	TAGAAAAATCAGGAGGTGT
BIP			
B1c	48	268-292	ACAAAAGAGGCAAAAAGTTCTATTG-
B2		324-346	CAGAATTTTTTATATCCGCATCG
F3	22	181-202	AAGTTACTTATATGGGAAAAGG
B3	23	361-383	GTGTTATCATCTTCTATAACAAC
F loop	14	222-235	CTGTAAAATTTTCA
B loop	15	296-310	AACACTTTAGTTTAT

LAMP=Loop mediated isothermal amplification assay, CaPV=*Capripoxvirus*

### Optimization of LAMP assay

Assay was optimized using different concentrations of external, internal primers and loop primers followed with concentration of betain (1, 1.5 and 2 M) and MgSO_4_ (1,2,4,6,8 and 10 mM) as well as at different temperature conditions. An intercalating dye (Picogreen, Molecular Probes, Invitrogen, Paisley, UK) and agarose gel-based electrophoresis were used to detect amplified DNA.

### Conventional PCR and quantitative PCR (qPCR) amplifications

Taq Man assay is highly sensitive quantitative method for detection of CaPVs [19]. Therefore, it was used for performance evaluation of LAMP assay. The primers and probe for the real-time PCR were designed which allowed the amplification of the target P32 gene sequences in all CaPVs [23]. The assay was performed on a real-time PCR machine (ABI 7500) using the TaqMan universal PCR master mix (Applied Biosystems). The reaction mixture contains 1 pmole forward primer, 0.5 pmol reverse primer and 0.25 pmole probe and 2 µl template to make reaction 20 ul. Conventional PCR was performed in a reaction mix containing 0.4 µM of each outer primer (F3 and B3) using high fidelity Fusion Taq mastermix (×2 x concentrations) with GC buffer in 20 μL reaction containing 2 μL of template.

### Limit of detection

To find out absolute copy number of virus, 35 ng/µl of quantified recombinant plasmid containing P32 gene was serially diluted 10-fold and used for amplification for both qPCR and LAMP. Serial dilution was used to plot the standard curve. The copy number of virus was calculated as number of copies = (amount [ng] × 6.022 × 10^23^)/(length [plasmid + insert] × 1 × 10^9^ × 660) [24].

### Analytical specificity

To evaluate the LAMP assay specificity, other closely related small ruminants virus positive isolates such as Orf virus and RNA viruses of ruminants such as bluetongue virus (BTV), Peste des petits ruminants virus (PPRV), and Foot-and-mouth disease virus (FMDV) were used. Different clinical samples suspected of CaPVs were also used for evaluation of the assay.

## Results

### Virus isolation

Various isolates that included vaccine strain of SPPV and PCR positive field samples of CaPV were adapted to primary lamb testis culture where they produced characteristic pox virus CPE in the cell. More than 50 clinical samples were tested from various outbreaks in the North India (Jammu and Kashmir, Haryana, and U.P). From four scab samples the CaPVs were successfully isolated; one from goat (CaPV/IND/2013/04) and three (CaPV/IND/2013/01, CaPV/IND/2013/02, CaPV/IND/2013/03) from sheep after three blind passages in cell culture (Table-2). CPE was characterized by rounding, tract formation, retraction and ballooning, nuclear vacuolation, chromatin formation and loss of continuity of the monolayer 3-4 days post-inoculation.

**Table-2:** Details of isolates tested using CaPV real time assay.

Sample ID	Origin source	Type of sample	Real time PCR results	LAMP results	Specie of origin
CaPV/IND/2013/01	Jammu and Kashmir	Scab sample	Positive	Positive	Sheep
CaPV/IND/2013/02	Jammu and Kashmir	Scab sample	Positive	Positive	Sheep
CaPV/IND/2013/03	Jammu and Kashmir	Scab sample	Positive	Positive	Sheep
CaPV/IND/2013/04	U.P	Scab sample	Positive	Positive	Goat

PCR=Polymerase chain reaction, LAMP=Loop mediated isothermal amplification assay, CaPV=*Capripoxvirus*

### Conventional PCR

Expected sized amplicon of 578 bp from P32 gene that were generated from vaccine strain of SPPV were further gel purified and cloned in pJET1.2 cloning vector. Recombinant plasmid DNAs were tested for confirmation of positivity by amplification using vector specific primer and gene specific primers that generated correct size products of 696 bp (578 bp + 118 bp) and 578 bp respectively. The partial amplicon of 578 bp generated during this study from the upstream end of P32 gene along with another amplicon of 581, which was generated in a related study from the downstream end of the gene [22] was used to generate full-length coding sequence of P32 gene from RF vaccine strain of SPPV. This contig sequence was submitted to Genbank and was assigned accession number KJ679574.

### Optimization of LAMP assay

**Figure-2:**
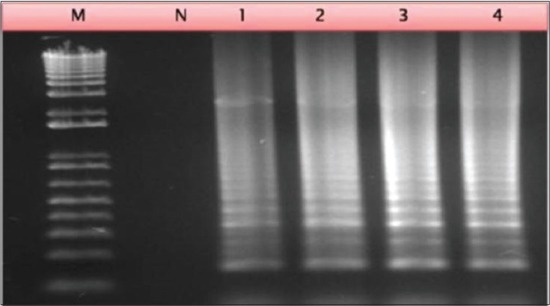
Agarose gel electrophoresis of loop mediated isothermal amplification assay products generated from sheeppox virus recombinant plasmid DNA using different time intervals. Lane 1: 30 min; Lane 2: 60 min; Lane 3: 90 min; Lane 4: 120 min.

**Figure-3:**
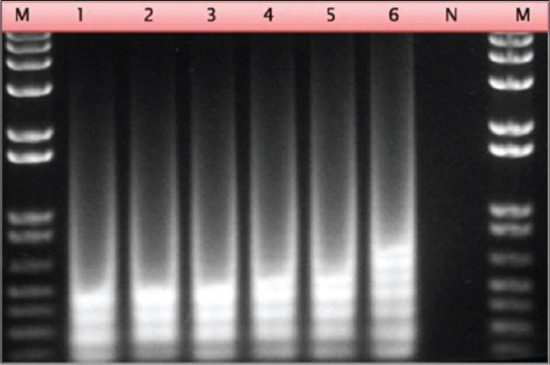
Agarose gel electrophoresis of loop mediated isothermal amplification assay products generated from sheeppox virus recombinant plasmid DNA using different temperatures. Lane 1-6: 60-65°C gradient.

All the variables described in material and methods were checked to optimize the LAMP assay. Taken together, the bright and distinct band patterns of different sizes were obtained at 65°C incubation in 60 min (Figures-2 and -3). The optimized assay conditions include incubation at 65°C for 1 h were optimized in a reaction master mix containing 1x isothermal amplification buffer (New England Biolabs, Hitchin, UK), 3 μM internal primers, 0.6 μM external primers, 1 mM MgSO4 (New England Biolabs, Hitchin, UK), 0.3 mM dNTPs (Sigma-Aldrich, Dorset, UK), 1 M betaine (Sigma-Aldrich, Dorset, UK), 16 U of Bst DNA polymerase (Warm start: New England Biolabs, Hitchin, UK). The positive LAMP reaction generated a ladder-like pattern with a set of bands of different sizes consisting of several inverted-repeat structures. LAMP assay was performed with and without loop primers targetting the P32 gene of CaPVs. The optimum yield for LAMP assay was obtained in 1 h but detection using color change product can be measured in ½ h only in presence of loop primers. In absence of loop primers, it requires considerably longer incubation.

### Specificity of LAMP primers

The viral DNA, as well as positive SPPV recombinant plasmid, was amplified using LAMP outer primers F3 and B3. A 203 bp amplified product matched with the predicted size of LAMP amplicon indicating primers are highly specific. A 203 bp base pair product was further gel purified and sequenced using big dye terminator method. The sequence obtained on BLAST analysis show 100% nucleotide identity with corresponding loci on P32 gene of CaPV. For further verification, amplified product was purified and used for re-amplification by LAMP which demonstrates LAMP products are from same template.

### Detection limit of LAMP assay

**Figure-4:**
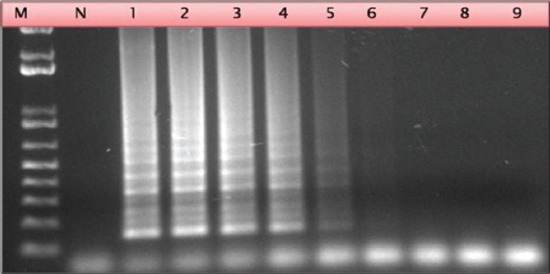
Agarose gel electrophoresis of loop mediated isothermal amplification assay products generated from sheeppox virus (SPPV) recombinant plasmid DNA. Lane 1: Negative control showing no amplification; Lanes 2-7: Serial 10 fold dilution series of SPPV recombinant plasmid DNA showing amplification until 10^–6^ dilution (7 pg).

**Figure-5:**
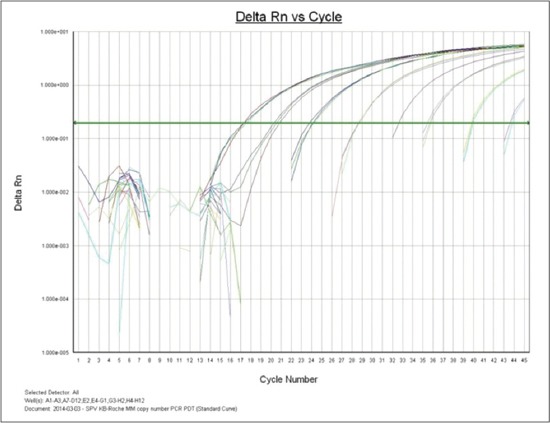
Real time amplification of serial 10 fold dilution of sheeppox virus recombinant plasmid DNA in TaqMan based assay.

**Figure-6:**
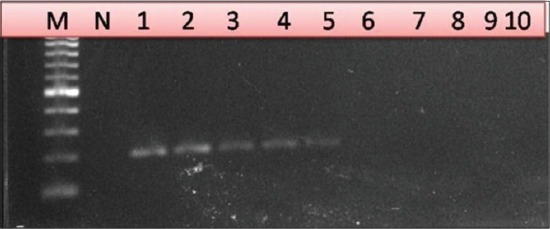
Agarose gel electrophoresis of 203 bp products generated from sheeppox virus (SPPV) recombinant plasmid DNA using loop mediated isothermal amplification assay outer primers of P32 gene. Lane 1: Negative control showing no amplification. Lanes 2-7: Serial 10 fold dilution series of SPPV recombinant plasmid DNA showing amplification until 10^–5^ dilution.

**Figure-7:**
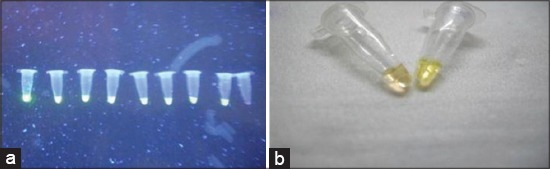
Limit of detection of *Capripoxvirus* loop mediated isothermal amplification (LAMP) assay determined using color change method by intercalating dye picogreen. (a) Serial 10 fold dilution of LAMP products under ultra-violet, (b) Color change by naked eye.

Serial dilutions of cloned recombinant plasmid containing P32 gene were used for evaluation of limit of detection of LAMP assay. LAMP dilutions were monitored both using agarose gel electrophoresis and color change of picogreen dye. For performance evaluation, detection limit was also measured by qPCR and conventional PCR using outer primers. Based on recombinant plasmid concentration, this assay can determine concentration of 7 pg of recombinant plasmid DNA and minimum of 100 copy number of virus particle whereas qPCR show 100 times more sensitivity with a detection limit of 70 fg or 0.07 pg (Figures-4 and -5). Conventional PCR results show 10 times lower sensitivity than LAMP assay using outer LAMP primers (Figure-6). Results showed distinct color by intercalating picogreen dye in serial 10 fold dilutions of LAMP products (Figure-7).

### Evaluation of assay

**Figure-8:**
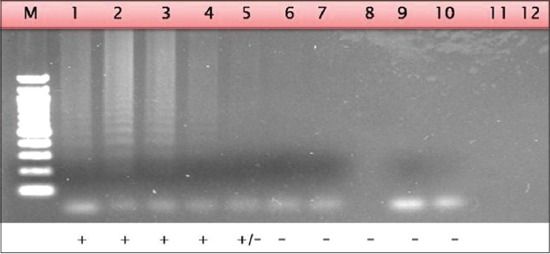
Agarose gel electrophoresis of clinical samples tested using loop mediated isothermal amplification assay. Lane 1: *Capripoxvirus* (CaPV)/IND/2013/02; Lane 2: CaPV/IND/2013/01; Lane 3: CaPV/IND/2013/04; Lane 4: CaPV/IND/2013/03; Lane 5: Weak Positive sample; Lane 6-10: Negative samples.

The LAMP assay was evaluated for its sensitivity and specificity using more than 50 clinical samples obtained from various outbreaks in North India (Jammu and Kashmir, Haryana and U.P). The assay showed high specificity and sensitivity in identification of clinical samples, which was comparable to other published CaPV specific assay [19]. The LAMP amplification of DNA purified from isolates which are listed in Table-2 is shown in Figure-8.

### Analytical specificity of assay

Orf virus, BTV, FMDV, and PPRV were tested and detected negative by CaPV specific LAMP assay. Lack of cross-reactivity with other viruses indicates that assay is highly specific for detection of CaPV DNA and does not show any cross reaction with other related viruses.

## Discussion

A fast, robust and reliable technique is the foremost requirement for detection of endemic diseases in countries such as India where one outbreak alone contributes to economic loss in millions of rupees. To monitor the disease at local level, there is urgent need for rapid diagnostic tool to identify the etiological agent which eventually helps in devising the prevention and control strategy. Molecular tools based on amplification of nucleic acids are well proven sensitive techniques capable of diagnosing any disease. SPPV and GPPV epizootics occur in several parts of India directly devastating livelihood of poor people [3,25]. Though virus isolation is considered as the gold standard but it suffers from the disadvantage of long incubation time (10-14 days). Molecular diagnostic tools like PCR and real-time PCR have been already described for CaPVs which are more rapid, sensitive and detection in real time [11-13,15-19,26-29]; however, they require more sophisticated instruments, skills, and highly validated platforms. This report describes a more convenient alternative LAMP assay which can detect capripoxviral pathogen infecting small ruminants. Due to its isothermal amplification (65°C) this technique can be performed in water bath or heating blocks which counter the cost of any equipment such as thermal cycler or real-time PCR. LAMP is highly specific due to four to six pair of primers targeting eight different genomic regions. There are several reports of LAMP assay targeting different diseases of livestock such as FMD, PPR, and BTV [30-32]. More recently, LAMP assays targeting conserved genes encoding the poly(A) polymerase small subunit (VP39) and P32 regions of CaPV genome using hydroxynaphthol blue (HNB) as an indicator dye have been described [21,24]. These described assays are from 3’ terminal end of P32 gene which is less conserved and have many polymorphic sites present particularly in Indian strains which may challenge the reliability of assay. In this study, thus LAMP assay targeting highly conserved region from 5’ terminal end of P32 gene was designed and optimized. The performance of assay was further evaluated using different clinical samples. This capripox LAMP assay is capable to detect as low as 100 copy number of viral particle which is 100 times less sensitive than qPCR having detection limit of just one copy number however, 10 times more sensitive than conventional PCR. Of the eight regions targeted by the LAMP oligonucleotides, six primers (F3, F1c, F Loop, B3, B Loop, and B2) were completely conserved across all of the available sequences in database, while only one nucleotide substitution was present in two primers F2 and B1c indicating test can be validated for many strains of CaPV.

LAMP assay is more rapid method than real time PCR consisting of ramp time and temperature with cycle number and costly thermal cycler. For any real-time PCR, it requires 75 min to generate a cycle threshold and determining real-time amplification whilst for a gel based it is more than 120 min. In this study, LAMP reaction can provide positive results in 30 min after incubation at 65°C and negative result defined not yielding any LAMP product after 60 min.

For designing of primers, Primer Explorer v4 software was used. The various sequence of SPPV, GPPV, and LSDV virus were aligned and uploaded in software. Default settings were unable to provide suitable primer pair due to rich AT content in this gene of CaPV, therefore various conditions and annealing temperatures were modified to obtain the suitable pair. This can be defined as inefficiency of software to generate primers for AT-rich sequences. Detection systems used for LAMP are (i) agarose gel electrophoresis; (ii) Magnesium pyrophosphate turbidity method [33], (iii) DNA-intercalating dye method, such as SYBR green [34], (iv) metal ion- binding fluorophore, such as calcein [35], and (v) metal ion-binding indicator dye such as HNB [36]. In this study, LAMP products were measured using color change method by intercalating dye picogreen which changes to green in the presence of amplified products. The accuracy was further measured by agarose gel electrophoresis, and it was found that samples having no change in color have no visible ladder like patterns. This method is more robust for positive result detection and more rapid than 1 h electrophoresis in gel assembly. Although only a limited number of clinical specimens were available for testing, this LAMP method was used successfully to detect and quantify SPPV in different types of specimen including tissues and cell culture derived materials. A single available field strain of GPPV was also tested positive in the assay; however, no sample of LSD was available for validation. The assay was designed using multiple sequences of P32 gene of CaPV isolates from different geographical origins hence the assay is supposed to be robust.

## Conclusion

This LAMP assay offers a potent tool for rapid identification of CaPV without the need for sequencing or any post-PCR processing. The LAMP assay described here has high sensitivity, specificity and reproducibility for simultaneous detection and quantitation of CaPV and can play significant role in its molecular epidemiology and surveillance studies.

## Authors’ Contributions

KB and SM: Drafted the manuscript. KB, AK, NSM, VK, TN, SM: Proof read the manuscript and provided the guidance. KB, AK, NSM, VK: Conducted the experiments. SM, NSM, AK: Provided reagents and material. All authors read and approved the final manuscript.
